# Discharge Follow-Up of Patients in Primary Care Does Not Meet Their Care Needs: Results of a Longitudinal Multicentre Study

**DOI:** 10.3390/nursrep14030180

**Published:** 2024-09-18

**Authors:** Noelia López-Luis, Cristobalina Rodríguez-Álvarez, Angeles Arias, Armando Aguirre-Jaime

**Affiliations:** 1 Doctoral Program in Medical and Pharmaceutical Sciences, Development and Quality of Life, University of La Laguna, 38200 Santa Cruz de Tenerife, Spain; alu0100754629@ull.edu.es; 2 Department of Preventive Medicine and Public Health, University of La Laguna, 38200 Santa Cruz de Tenerife, Spain; angarias@ull.edu.es; 3 Health Care Research Support Service, Nurses Association of Santa Cruz de Tenerife, 38001 Santa Cruz de Tenerife, Spain; armagujai@gmail.com

**Keywords:** continuity of patient care, patient discharge, primary healthcare, patient transfer

## Abstract

Adequate coordination between healthcare levels has been proven to improve clinical indicators, care costs, and user satisfaction. This is more relevant to complex or vulnerable patients, who often require increased care. This study aims to evaluate the differences between hospital discharge follow-up indicators, including number of general practitioners’ (GPs) and community nurses’ (CNs) consultations, presentiality of consultations, type of first post-discharge consultation, and time between hospital discharge and first consultation. Vulnerable and non-vulnerable patients were compared. A longitudinal retrospective study was carried out in the north of Tenerife on the post-discharge care of patients discharged from the Canary Islands University Hospital (Spanish acronym HUC) between 1 January 2018 and 31 December 2022. The results obtained show deficiencies in the care provided to patients by primary care (PC) after being discharged from the hospital, including delayed first visits, low presentiality of those visits that were less frequent even with increased patient complexity, scarce first home visits to functionally impaired patients and delays in such visits, and a lack of priority visits to patients with increased follow-up needs. Addressing these deficiencies could help those most in need of care to receive PC, thus reducing inequalities and granting equal access to healthcare services in Spain.

## 1. Introduction

Over the last few decades, healthcare integration has become a priority for many healthcare systems, particularly in addressing chronic health issues involving multiple professionals and services. Coordination between healthcare levels refers to the degree of connection needed to provide continuous and synchronised care to a patient [1].

Continuity of care has been defined as the degree to which patients experience a series of healthcare events as coherent and interconnected over time [2]. It is associated with increased user satisfaction [3,4,5,6], better perceived quality of life [7], greater use of preventive services [3,6,7,8,9], higher adherence rates to treatment [5,6,9], and lower hospital admission rates [10].

Continuity of care is challenging due to the non-coordinated levels, especially post-discharge for complex or vulnerable patients needing more care. Vulnerable patients have additional personal, psychosocial, or health circumstances that increase their risk and fragility and require special care by the PC team [11].

Frailty assessment at hospital discharge has shown a significant correlation with non-elective readmission, major adverse cardiac and cerebral events, and post-stroke disability at 3 months, highlighting its importance in post-hospital care planning [12]. However, thorough cognitive function assessments and the consideration of age-specific needs are crucial when planning home- and community-based services [13].

Regarding post-hospital discharge follow-up and socioeconomic vulnerability, a Danish study linked lower socioeconomic status with increased cardiovascular mortality in patients discharged after atrial fibrillation [14]. Additionally, two US studies found higher mortality and readmission rates among patients with lower socioeconomic status [15,16].

This study hypothesises that the most vulnerable patients with the greatest need of care at discharge do not receive priority care that meets their greater needs. This study aims to compare post-discharge follow-up indicators, such as the number of GP and CN consultations, presentiality of consultations, type of first consultation after discharge, and time elapsed from hospital discharge to these first consultations between vulnerable and non-vulnerable patients. The goal is to identify potential deficits in the organisation of the PC system; resolving these deficits would make the system more inclusive and equitable by matching the post-discharge follow-up to the patient’s vulnerability level.

## 2. Materials and Methods

### 2.1. Study Design

A retrospective study was conducted across 23 health centres in Tenerife, focusing on the post-discharge care of patients released from the HUC between 1 January 2018 and 31 December 2022. Inclusion criteria were as follows: having been classified during their hospital stay as fragile patients according to the Care Integration and Coordination Subprogram (acronym in Spanish SPICA), which is dedicated to care coordination with the participation of CNs [17]. In addition, patients must have been cared for by the PC public healthcare services of Tenerife after hospital discharge, have a PC clinical report, and not be deceased at the time of data collection.

### 2.2. Data Collection Instruments

The information about the patients included in this study according to selection criteria was collected from DRAGO-AP computerised medical records using an anonymised procedure. Data obtained were sex and age, assigned health centre, diagnosis at admission to the HUC, hospital admission and discharge dates, and whether the patient was deceased at the time of data collection.

The patient’s complexity level was obtained using the Kaiser–Permanente risk pyramid [18], cognitive status assessments were performed using Pfeiffer test [19], functional status was obtained using Katz [20,21] and Lawton–Brody [22] before admission and at discharge, and falls and risk of falls were obtained using Downton [23,24]. Use of orthopaedic or prosthetic devices before and after admission was also obtained. As for socio-familial characteristics, the following data were obtained: type of family, family life, number of cohabitants, prior carer and carer at discharge, sex of the carer, home help, and having processed the application included in the Dependency Act [25]. Medical records were also used to obtain post-discharge follow-up at 6 months according to the number of GP and CN consultations, the presentiality of consultations, date and type of first consultation, consultation with the centre’s social worker (SW), visits to the PC or hospital A&E department, and hospital readmissions.

According to this study’s hypothesis, it is crucial to identify the patient most in need of follow-up after hospital discharge by PC. This definition should consider the patient most in need of the said follow-up to be those with the worst biological, psychological and social conditions, also considering ease of patient classification using the information normally available in the clinical records. Being over 80 or having a level of high-risk complexity or high complexity, according to Kaiser’s pyramid, is considered a biological condition of priority for follow-up. Besides fulfilling either of the two said conditions, the patient should also present one of the following impairments: deterioration of functional state at discharge compared with before admission according to the Katz index or the Lawton and Brody scale or need to use orthopaedic or prosthetic devices at discharge. The suffering of deterioration in cognitive status after discharge compared with before admission according to the Pfeiffer test was considered a psychological condition of priority for follow-up. The socio-familial condition considered of priority for follow-up involved the patient being in at least one of the following situations: living alone, being in an elderly couple with no other cohabitant, being a single parent with children, being widowed, or lacking a carer at discharge despite needing it. The patient with the greatest need for follow-up by PC after hospital discharge would be one who meets these three conditions.

### 2.3. Population and Sample

A sample of 387 patients meeting the inclusion criteria were randomly selected, giving this study a potency of 95% in comparison of proportions, relative frequencies and medians, with a sensitivity for the detection of relevant differences of at least 10% and 5 points in medians with dispersions of 15 points.

### 2.4. Statistical Analysis

The characteristics of the patients are summarised with the absolute and relative frequencies of their component categories if the variable is nominal, and with the median (minimum–maximum) if it is of a numerical scale due to its non-normal distribution verified with the Kolmogorov–Smirnov test. The estimation of the significance of the differences in nominal variables is determined with Pearson’s Chi2 test and the scale between more than two groups is determined with the Kruskal–Wallis H test and post hoc Mann–Whitney U test. The strength and direction of association between scale variables are estimated with the Spearman correlation coefficient and the adjustment of simple linear regression models.

Multivariable linear regression models were adjusted with consultations as the effect variable and all those variables that achieved a statistical significance of at least *p* ≤ 0.10 in the difference in consultations as potential explanatory factors. In the case of dichotomous effects, the models that are adjusted are multivariable binary logistics with the same criterion was used for selecting explanatory factors. The adjustment strategy was that of full models, performed by backward steps with the statistical significance or Wald criterion. The number of patients classified as vulnerable permits the use of up to 5 potentially explanatory factors according to the Hosmer–Lemeshov rule. Hypothesis contrast tests were bilateral at a statistical significance level of *p* ≤ 0.05, except in the case of regression models in order to rule out interactions between factors. The involved calculations and estimates were executed with IBM Co.^®^ SPSS 25.0™.

## 3. Results

The study consisted of 387 patients. Their main characteristics are shown in Table 1. We found a higher proportion of women, a high level of complexity among the included patients, and a high percentage of functional deterioration at discharge. As for family characteristics, most were nuclear families and most carers at discharge resulted to be family members. We also found that carers at discharge were mainly women (74%), previous falls reached a considerable proportion (58%), and risk of falls was very high (58%). No differences between sex were observed in the number of consultations with the GP or CN: nine (1–58) in men vs. ten (2–66) in women with the GP (*p* = 0.087) and seven (0–719) in men vs. eight (0–76) in women with the CN (*p* = 0.535), but a direct correlation was found between patient’s age and number of consultations, both with the GP (ῤ = 0.146, *p* < 0.001) and the CN (ῤ = 0.153, *p* < 0.001).

Table 2 shows the follow-up required for the patient according to their complexity classification. The number of consultations, both with the GP and with the CN, increases with the patients’ complexity level, and no statistically significant differences were obtained in the other follow-up indicators. A comparison of differences in the number of consultations by complexity level between the GP and the CN showed that this number is higher in lower-risk patients with chronic conditions and with a GP (*p* = 0.011). On the other hand, the percentage of first home visits among those who showed worsened functionality measured with the Katz index is much higher in the CN than in the GP.

Table 3 shows the relationship between the functional and cognitive changes from admission to the discharge and follow-up of the patient. Regarding the functional deterioration according to the Katz index, it was found that the main type of first consultation with the GP is generally a virtual consultation, both for patients who improve and for those who deteriorate, whereas among those whose status remains unchanged, it is a first visit to the centre. Regarding the CN, the first consultation of those who deteriorate is home consultation; for those whose status remains unchanged, it is a visit to the centre; and for those who improve, it is shared between a home consultation and a visit to the centre. Among those who deteriorate, a gradient of fewer home visits and mostly online consultations with the GP is shown, whereas an inverse gradient is shown in the case of the CN: more home visits and fewer online consultations with deteriorating patients. Most home visits to deteriorating patients are carried out by the CN (43% vs. 11%, *p* < 0.001). As for changes in instrumental activities according to the Lawton–Brody scale, it was found that patients who improve are seen earlier than those who deteriorate. Finally, regarding change in cognitive state, patients whose status remains unchanged are most frequently seen in-person by their GP, followed by those who improve and, lastly, those who deteriorate. A third of the patients suffering from deteriorations of their cognitive state are not seen in-person by their GP at 6 months.

Table 4 provides tracking indicators related to biological, psychic, socio-family, and global needs. Regarding biological condition, more GP and CN visits were found to be associated with patients with the greatest needs. The CN also pays more home visits to those patients and sees them mostly in-person. In addition, the percentage of in-person visits performed by the CN is higher compared to the GP whether they worsen or not. No difference is observed in any indicator for the psychic need. For the socio-family condition, higher numbers of GP in-person consultations were observed among those fulfilling this condition, but with marginal statistical significance. As for the global need, a greater number of GP and CM visits was observed among those fulfilling this condition, who are also more often seen in-person by the CN.

Table 5 shows the results of the regression model adjustments. The first block shows the results of the linear regression models, with effects seen on the number of visits to the GP and CN, and a reduction in the number of days between discharge and first visit to the CN. The second block shows the results of the binary logistic regression models with in-person visit to the GP and CN as effects. The GP consultation increases on average by 3.8 if the patient has been readmitted, by 3.8 if the patient has visited the PC A&E department, and by 1.9 for each rise in complexity level. The number of CN consultations increases on average by 4.7 for each rise in complexity level and by 0.09 for each additional day of hospital stay. The time elapsed between discharge and first visit to the CN decreases on average by 7.6 days whenever the patient fulfils the biological condition of priority follow-up. The possibility of an in-person visit to the GP increases on average by 8.2 if the cognitive status of the patient remains unchanged. The possibility of an in-person visit to the CN increases on average by 3.3 whenever the patient fulfils the biological condition of a greater need of follow-up.

## 4. Discussion

The results of this study confirm the initial hypothesis that patients with the greatest needs of follow-up at hospital discharge do not receive priority follow-up in PC. Despite the patients with the greatest needs of global follow-up presenting a slight increase in number of visits and having more in-person consultations with the CN, most follow-up indicators do not show a positive discriminatory difference in this area.

This study uses ubiquitous classifications and ratings as labels for patients most in need of follow-up, including one created by the authors. The decision to create this classification is supported by the absence of a label for patient follow-up need after hospital discharge. In our study, we have used criteria commonly associated with frailty, covering factors that influence vulnerability and that are individually linked to worse health outcomes and higher readmission rates, as indicators of follow-up need [26,27,28]. This definition addresses the biological, psychic and socio-family dimensions of health status inherent in the patient-centred care approach as a paradigm of PC [29,30] and meets the criterion of ease of use and care utility. One might argue against the spuriousness of combining patient status indicators to classify them as in need of follow-up when the direct use of such indicators would be sufficient, but the emergent property of Bertalanffy’s biological systems [31] conceptually supports it.

Our sample is representative of particularly complex patients given the use of selection criteria that employ SPICA, as pointed out by García-Hernández [17], so their functional deterioration is greater than is observed in other studies, where only a third suffered deterioration [32]. Regarding carers’ characteristics, the majority were found to be women, in agreement with other studies carried out in Spain [31,32,33,34]. Moreover, most carers were family members, which could be explained by the deeply rooted Spanish tendency towards family solidarity and the limitation of public resources dedicated to dependency care [35,36].

The relationship found between complexity and follow-up coincides with recent studies carried out in Spain, where the most complex patients according to the Kaiser pyramid accumulated a greater number of visits in PC and showed more frequent visits to the GP than to the CN [37], a tendency maintained in this study after hospital discharge. However, this study found that more complex patients do not receive a visit closer to discharge, nor are they seen in person more often. Furthermore, their in-person visits to the GP decrease as their complexity increases.

In the case of patients with access difficulties, home visits are crucial to ensure equity in the care [38,39]. It is thus surprising that the results showed that, in patients whose functionality deteriorated, the GP undertook fewer first home visits. However, the opposite results were derived on the part of the CN. A qualitative study found a series of challenges to home care performed by nurses, such as difficult instances, economic problems, professional barriers, social difficulties, and bureaucratic tension [40]. This result requires an analysis to identify the specific barriers blocking home visits in our environment.

Regarding the change in instrumental activities, we find that, paradoxically, those who improve are seen sooner, while it is known that functional deterioration is the main determinant of morbidity and mortality and of the consumption of health and social resources after hospital discharge [41,42,43].

The follow-up indicators of the most needy patients reveal that they are not attended to as a priority. The lack of differences in post-discharge follow-up points to the absence of discrimination in the setting of priority of care for the most fragile patients.

Here, the number of GP visits increases whenever the patient is readmitted, in agreement with the results obtained by another Spanish study [27]. Consultation frequency in general also increases in patients who visit the PC A&E department and who show greater complexity. The second seems feasible, as a rise in CN consultations was observed with the increase in the complexity level of the patient. Regarding CN visits, it was found that patients fulfilling the biological conditions setting priority for follow-up are seen sooner and encounter more possibilities for in-person consultations. Such evidence could serve in establishing predictors in the management of consultations and priority of care.

The high number of patients who are not evaluated in person by their GP or CN in the 6 months following discharge is alarming because, through these visits, the evolution of their health is evaluated, possible complications are detected, and the patient’s environment, support network, and barriers they face are addressed. Patients in our environment have associated the lack of in-person visits with a lower quality of care, described as a loss of effectiveness, and a lack of trust and security in care [44].

Overall, our results point to an inequity in access to health services, a persistent challenge that goes against the preferential distribution of resources and attention to the neediest, thus confirming Tudor Hart’s inverse care law [45]. A comparison of this study’s results with those for other countries is hindered by the considerable differences between healthcare systems.

Our study presents some important limitations. Firstly, its observational nature is an issue; however, the needs for post-discharge follow-up arise prior to the follow-up, so associations have a natural cause–effect temporality. Another limitation is that the peculiarity of SPICA patients, with greater fragility and subjected to a special coordination process between levels of care, makes it difficult to extrapolate our results to the general population, for whom follow-up would reveal even worse indicators. The third limitation is the exclusion of patients who had died at the time of data collection, as the software program did not allow access to that information whenever a lack of appropriate post-hospital discharge follow-up was among the possible causes of death. Although unsolved, this could only lead to worse results than those obtained.

Among the strengths of this study are its multicentric character, the large volume of patients included, and the variety of biopsychosocial variables considered. Additionally, we might note the attention paid to aspects related to family and carers at discharge that have been insufficiently studied in previous works.

According to our results and considering its limitations, the initial hypothesis that patients with greater needs of post-hospital discharge follow-up do not receive priority follow-up in PC cannot be disregarded.

In conclusion, the results show deficiencies in the care provided to patients in PC following hospital discharge, particularly in the delay of visits after discharge, the low presentiality of visits (even lower with increased patient complexity), the number of first in-person visits to patients with impaired functionality and delays in such visits, and the lack of priority visits to patients with a greater need of follow-up. Considering these deficiencies and addressing them would help PC to reach those more in need of it, thus reducing inequalities and granting fair access to healthcare services in Spain.

Future research is required that considers not only quantitative indicators but also qualitative aspects and a global perspective of patient care after hospital discharge.

## Figures and Tables

**Table 1:** Characteristics of patients participating in the association study between vulnerability of patients discharged from hospital and their follow-up in PC.

Characteristic	Categories	N Value (%) or Median (Min–Max.)
Sex	Man	151 (39)
	Woman	236 (61)
Age	Years	76 (23–97)
Complexity	Chronic	87 (25)
	High risk	166 (48)
	High complexity	83 (24)
Hospital stay	Days	17 (1–279)
Use of orthopaedic or prosthetic devices at discharge	Wheelchair	86 (29)
	Shower chair	63 (17)
	Walking frame	183 (48)
	Cane	28 (7)
	Crutches	35 (9)
	Articulated bed	21 (6)
	Anti-bedsore mattress	4 (1)
Change in functional status at discharge according to Lawton-Brody scale	Unchanged	108 (54)
	Deteriorate	89 (48)
	Improve	2 (1)
Change in functional status at discharge according to Katz index	Unchanged	99 (27)
	Deteriorate	267 (72)
	Improve	6 (2)
Change in cognitive status at discharge according to Pfeiffer scale	Unchanged	74 (64)
	Deteriorate	33 (29)
	Improve	8 (7)
Family type	Lives alone	18 (5)
	Nuclear	303 (79)
	Equivalent	41 (11)
Family life cycle	Contraction	168 (54)
	Death	107 (34)
Carer at discharge	Does not have	5 (1)
	Family member	291 (79)
	Cohabitant	214 (67)
Resources at discharge	Private home care services	69 (18)
	Council home care services	135 (36)
	Dependency Act	100 (27)
Greater complexity at discharge due to biological condition	277 (72)	
Greater complexity at discharge due to psychological condition	291 (75)	
Greater complexity at discharge due to socio-familial condition	196 (51)	
Greater complexity at discharge due to the three latter conditions	143 (38)	

**Table 2:** Relationship between complexity and follow-up indicators after hospital discharge.

Follow-Up Indicators		Complexity According to Kaiser Pyramid N (%) or Median (Min–Max)			*p* Value
		Lower Risk	High Risk	High Complexity	
Indicator	Categories	87 (26)	166 (50)	83 (25)	---
Number of consultations with GP	Frequency	9 (2–29)	10 (1–29)	12 (2–66)	0.002 ^1^
Number of consultations with CN	Frequency	6 (0–62)	8 (0–76)	10 (0–72)	0.001 ^1^
First visit to GP	Days since discharge	4 (0–126)	4 (0–50)	5 (1–35)	0.343 ^1^
First visit to CN	Days since discharge	4 (0–126)	3 (0–183)	5 (0–171)	0.064 ^1^
Type of first consultation with GP	Virtual	32 (38)	53 (33)	28 (35)	0.347 ^2^
	Phone	21 (25)	47 (29)	20 (25)	
	Health centre	26 (31)	39 (24)	26 (33)	
	Home	5 (6)	21 (13)	5 (6)	
Type of first consultation with CN	Virtual	7 (9)	15 (9)	9 (11)	0.696 ^2^
	Phone	21 (27)	45 (28)	22 (27)	
	Health centre	24 (31)	36 (22)	24 (30)	
	Home	26 (33)	67 (41)	26 (32)	
In-person consultations with GP	No	11 (13)	32 (19)	20 (25)	0.164 ^2^
	Yes	73 (87)	134 (81)	61 (75)	
In-person consultations with CN	No	11 (13)	10 (6)	6 (7)	0.180 ^2^
	Yes	76 (87)	155 (94)	77 (93)	
Consultations with SW	No	61 (70)	115 (71)	52 (63)	0.419 ^2^
	Yes	26 (30)	48 (29)	31 (37)	

^1^—Compared with Kruskal–Wallis H test. ^2^—Compared with Pearson’s Chi-square test.

**Table 3:** Relationship between functional and cognitive status of the patient and follow-up received.

Follow-Up Indicators		Functional/Cognitive Status N (%) or Median (Minimum–Maximum)											
		According to Katz Index				According to Lawton–Brody Scale				According to Pfeiffer Scale			
Indicator	Categories	Deteriorate	Unchanged	Improve	*p* Value	Deteriorate	Unchanged	Improve	*p* Value	Deteriorate	Unchanged	Improve	*p* Value
GP consultations	Frequency	9 (1–32)	10 (1–58)	14 (3–27)	0.292 ^1^	10 (2–32)	10 (1–58)	10 (7–13)	0.966 ^1^	11 (3–27)	10 (2–29)	11 (3–26)	0.997 ^1^
CN consultations	Frequency	8 (0–76)	7 (0–71)	8 (0–27)	0.714 ^1^	8 (0–76)	8 (0–71)	9 (8–9)	0.338 ^1^	6 (0–28)	8 (0–72)	4 (0–14)	0.083 ^1^
First consultation with GP	Days since discharge	5 (0–126)	5 (0–83)	5 (1–8)	0.927 ^1^	4 (0–126)	5 (0–83)	3 (2–3)	0.049 ^1^	5 (0–18)	5 (0–126)	6 (0–11)	0.708 ^1^
First consultation with CN	Days since discharge	4 (0–182)	4 (0–183)	7 (0–29)	0.340 ^1^	3 (0–126)	4 (0–159)	1 (0–2)	0.029 ^1^	4 (0–171)	4 (0–126)	6 (0–27)	0.690 ^1^
First consultation with GP	Virtual	97 (38)	25 (26)	3 (60)	0.000 ^2^	35 (41)	36 (35)	1 (50)	0.113 ^2^	9 (28)	25 (38)	2 (25)	0.090 ^2^
	Phone	82 (32)	18 (19)	1 (20)		22 (26)	21 (20)	0 (0)		17 (53)	17 (27)	4 (50)	
	Health centre	48 (19)	46 (48)	1 (20)		17 (20)	37 (36)	0 (0)		5 (16)	23 (32)	2 (25)	
	Home	27 (11)	6 (6)	0 (0)		11 (13)	9 (9)	1 (50)		1 (3)	7 (10)	0 (0)	
First consultation with CN	Virtual	23 (9)	9 (10)	0 (0)	0.000 ^2^	10 (12)	10 (10)	1 (50)	0.724 ^2^	2 (7)	7 (10)	1 (14)	0.597 ^2^
	Phone	72 (27)	23 (24)	1 (20)		17 (20)	26 (26)	1 (50)		12 (49)	21 (30)	0 (0)	
	Health centre	53 (21)	43 (45)	2 (40)		22 (26)	29 (28)	0 (0)		6 (20)	21 (30)	3 (43)	
	Home	104 (43)	20 (21)	2 (40)		36 (42)	37 (36)	0 (0)		10 (33)	21 (30)	3 (43)	
In-person consultations with GP	No	52 (20)	19 (19)	1 (17)	0.889 ^2^	12 (14)	25 (24)	0 (0)	0.079 ^2^	10 (32)	8 (11)	20 (18)	0.027 ^2^
	Yes	210 (80)	80 (81)	5 (83)		76 (86)	81 (76)	2 (100)		21 (68)	66 (89)	6 (75)	
In-person consultations with CN	No	22 (8)	12 (12)	1 (17)	0.256 ^2^	6 (7)	12 (11)	0 (0)	0.289 ^2^	5 (15)	5 (7)	2 (25)	0.159 ^2^
	Yes	245 (92)	87 (88)	5 (83)		83 (93)	96 (89)	2 (100)		28 (85)	69 (93)	6 (75)	
Consultations with SW	No	184 (69)	70 (71)	3 (50)	0.532 ^2^	52 (59)	75 (70)	2 (100)	0.161 ^2^	24 (75)	48 (66)	6 (75)	0.596 ^2^
	Yes	82 (31)	28 (29)	3 (50)		36 (41)	32 (30)	0 (0)		8 (25)	25 (34)	2 (25)	

^1^—Compared with Kruskal–Wallis test. ^2^—Compared with Chi-square test.

**Table 4:** Relationship between greater patient’s need of post-hospital discharge follow-up and post-hospital discharge follow-up received in PC.

Follow-Up Indicators		Greater Need of Post-Hospital Discharge Follow-Up Due to His/Her Condition:											
		Biological			Psychological			Socio-Familial			Global		
Indicator	Categories	No	Yes	*p* Value	No	Yes	*p* Value	No	Yes	*p* Value	No	Yes	*p* Value
Consultations with GP	Frequency	8 (1–29)	10 (1–66)	0.001 ^1^	9 (1–66)	11 (2–58)	0.263 ^1^	9 (1–32)	10 (2–66)	0.945 ^1^	9 (1–32)	10 (2–66)	0.033 ^1^
Consultations with CN	Frequency	5 (0–70)	8 (0–76)	0.000 ^1^	8 (0–76)	6 (0–58)	0.176 ^1^	7 (0–76)	8 (0–72)	0.154 ^1^	6 (0–76)	9 (0–72)	0.000 ^1^
First visit to GP	Days since discharge	4 (0–83)	5 (0–126)	0.230 ^1^	5 (0–126)	4 (0–50)	0.141 ^1^	4 (0–126)	5 (0–95)	0.531 ^1^	4 (0–126)	5 (0–95)	0.149 ^1^
First visit to CN	Days since discharge	4 (0–183)	4 (0–171)	0.085 ^1^	4 (0–183)	3 (0–122)	0.597 ^1^	4 (0–182)	4 (0–183)	0.626 ^1^	4 (0–182)	4 (0–166)	0.262 ^1^
Type of first consultation with GP	Virtual	36 (35)	94 (36)	0.394 ^2^	96 (35)	34 (37)	0.832 ^2^	66 (37)	64 (34)	0.532 ^2^	85 (37)	45 (32)	0.786 ^2^
	Phone	29 (28)	76 (28)		78 (28)	27 (30)		54 (30)	51 (27)		63 (27)	42 (30)	
	Health centre	36 (31)	67 (25)		78 (28)	21 (23)		42 (23)	57 (30)		59 (26)	40 (29)	
	Home	6 (6)	29 (11)		26 (9)	9 (10)		18 (10)	17 (9)		22 (10)	13 (9)	
Type of first consultation with CN	Virtual	5 (5)	28 (10)	0.008 ^2^	24 (9)	9 (10)	0.107 ^2^	18 (10)	15 (8)	0.576 ^2^	19 (9)	14 (10)	0.827 ^2^
	Phone	24 (25)	78 (29)		76 (27)	26 (30)		47 (26)	55 (29)		59 (26)	43 (30)	
	Health centre	38 (40)	61 (22)		84 (30)	15 (17)		44 (24)	55 (29)		63 (28)	36 (25)	
	Home	28 (30)	105 (39)		95 (34)	38 (43)		69 (39)	34 (34)		82 (37)	51 (34)	
In-person consultations with GP	No	19 (18)	55 (20)	0.581 ^2^	47 (16)	27 (29)	0.824 ^2^	44 (23)	30 (15)	0.050 ^2^	51 (22)	23 (16)	0.175 ^2^
	Yes	89 (82)	219 (80)		241 (84)	67 (71)		144 (77)	164 (84)		186 (78)	122 (84)	
In-person consultations with CN	No	19 (17)	16 (6)	0.000 ^2^	24 (8)	11 (11)	0.347 ^2^	22 (12)	13 (7)	0.097 ^2^	29 (12)	6 (4)	0.009 ^2^
	Yes	91 (83)	260 (94)		266 (92)	85 (89)		169 (88)	182 (93)		212 (88)	139 (96)	
Consultations with SW	No	83 (75)	182 (66)	0.084 ^2^	200 (69)	65 (69)	0.973 ^2^	125 (66)	140 (72)	0.231 ^2^	165 (69)	100 (69)	0.988 ^2^
	Yes	27 (24)	92 (34)		90 (31)	29 (31)		64 (34)	55 (28)		74 (31)	45 (31)	

^1^—Compared with Kruskal–Wallis test. ^2^—Compared with Chi-square test.

**Table 5:** Results of multivariable linear and logistic regression models’ adjustments with frequency of visits to GP and CN and presentiality of consultations as effects.

Mode	It Happens	Because the Patient…	In Consultations or Times [B(CI95%) or OR(CI95%)]	*p* Value
Linear	increase in consultations with GP ^1^	has had readmissions	3.814 (1.202–6.426)	0.004
		has urgent PC consultations	3.807 (1.930–5.685)	<0.001
		complexity increases by one level	1.912 (0.790–3.034)	0.001
	increase in consultations with CN ^2^	complexity increases by one level	4.714 (3.810–5.618)	<0.001
		increases his hospital stay by a day	0.093 (0.039–0.146)	0.001
	reduction in days between discharge and first visit to CN	has greater need of follow-up due to biological condition	7.676 (1.542–13.810)	0.014
Logistic	in-person visit to GP ^3^	cognitive status changes ^4^		
		for the better	ref	
		does not change	8.250 (3.961–17.184)	0.000
		for the worse	2.100 (0.989–4.459)	0.053
	in-person visit to CN ^5^	has greater need of follow-up due to biological condition	3.393 (1.674–6.878)	0.001

^1^—Converges at fourth iteration not retaining age, complexity, hospital stay, higher need of follow-up due to biological, socio-familial, and global condition. ^2^—Converges at third iteration not retaining age, greater need of follow-up due to biological and global condition. ^3^—Converges at second iteration not retaining urgent consultations in PC and greater need of follow-up due to socio-familial condition. ^4^—Valued as change in Pfeiffer scale between status prior to admission and at hospital discharge. ^5^—Converges at third iteration not retaining greater need of follow-up due to socio-familial and global condition.

## Data Availability

The data presented in this study are available on request from the corresponding author.

## Public Involvement Statement

There was no public involvement in any aspect of this research.

## Guidelines and Standards Statement

This manuscript was drafted against the Strengthening the Reporting of Observational Studies in Epidemiology (STROBE) Statement.

## References

[1] Starfield B. (2002). Coordinación de La Atención En Salud. Atención Primaria.

[2] Haggerty J.L., Reid R.J., Freeman G.K., Starfield B.H., Adair C.E., McKendry R. (2003). Continuity of Care: A Multidisciplinary Review. BMJ.

[3] Saultz J.W., Albedaiwi W. (2004). Interpersonal Continuity of Care and Patient Satisfaction: A Critical Review. Ann. Fam. Med..

[4] Gulliford M.C., Naithani S., Morgan M. (2007). Continuity of Care and Intermediate Outcomes of Type 2 Diabetes Mellitus. Fam. Pract..

[5] O’Malley A.S. (2004). Current Evidence on the Impact of Continuity of Care. Curr. Opin. Pediatr..

[6] Guthrie B., Brampton S., Wyke S. (2000). Does Continuity in General Practice Really Matter? Commentary: A Patient’s Perspective of Continuity. BMJ.

[7] Hänninen J., Takala J., Keinänen-Kiukaanniemi S. (2001). Good Continuity of Care May Improve Quality of Life in Type 2 Diabetes. Diabetes Res. Clin. Pract..

[8] Cabana M.D., Jee S.H. (2004). Does Continuity of Care Improve Patient Outcomes. J. Fam. Pract..

[9] Gray D.P., Evans P., Sweeney K., Lings P., Seamark D., Seamark C., Dixon M., Bradley N. (2003). Towards a Theory of Continuity of Care. J. R. Soc. Med..

[10] Facchinetti G., D’Angelo D., Piredda M., Petitti T., Matarese M., Oliveti A., De Marinis M.G. (2020). Continuity of Care Interventions for Preventing Hospital Readmission of Older People with Chronic Diseases: A Meta-Analysis. Int. J. Nurs. Stud..

[11] Riquelme-Heras H., Gómez-Gómez C., Gutiérrez-Herrera R., Martínez-Lazcano F., Sierra-Ayala I. (2016). Criterios para Identificar Pacientes Vulnerables en Atención Primaria. Rev. Cuba. Med. Gen. Integral.

[12] He H., Liu M., Li L., Zheng Y., Nie Y., Xiao L.D., Li Y., Tang S. (2024). The Impact of Frailty on Short-Term Prognosis in Discharged Adult Stroke Patients: A Multicenter Prospective Cohort Study. Int. J. Nurs. Stud..

[13] Yu Y., Zhang J., Petrovic M., Zhang X., Zhang W.-H. (2024). Utilization of Home- and Community-Based Services among Older Adults Worldwide: A Systematic Review and Meta-Analysis. Int. J. Nurs. Stud..

[14] Hagengaard L., Andersen M.P., Polcwiartek C., Larsen J.M., Larsen M.L., Skals R.K., Hansen S.M., Riahi S., Gislason G., Torp-Pedersen C. (2021). Socioeconomic Differences in Outcomes after Hospital Admission for Atrial Fibrillation or Flutter. Eur. Heart J. Qual. Care Clin. Outcomes.

[15] Hartline J., Cosgrove C.T., O’Hara N.N., Ghulam Q.M., Hannan Z.D., O’Toole R.V., Sciadini M.F., Langhammer C.G. (2024). Socioeconomic Status Is Associated with Greater Hazard of Post-Discharge Mortality than Race, Gender, and Ballistic Injury Mechanism in a Young, Healthy, Orthopedic Trauma Population. Injury.

[16] Man S., Bruckman D., Tang A.S., Uchino K., Schold J.D. (2021). The Association of Socioeconomic Status and Discharge Destination with 30-Day Readmission after Ischemic Stroke. J. Stroke and Cerebrovasc. Dis..

[17] García-Hernández M., González de León B., Barreto-Cruz S., Vázquez-Díaz J.R. (2022). Multicomponent, High-Intensity, and Patient-Centered Care Intervention for Complex Patients in Transitional Care: SPICA Program. Front. Med..

[18] Canarian Health Service (2015). Servicio Canario de la Salud. Estrategia de Abordaje a la Cronicidad en la Comunidad Autónoma de Canarias.

[19] Pfeiffer E. (1975). A Short Portable Mental Status Questionnaire for the Assessment of Organic Brain Deficit in Elderly Patients. J. Am. Geriatr. Soc..

[20] Katz S., Downs T.D., Cash H.R., Grotz R.C. (1970). Progress in Development of the Index of ADL. Gerontologist.

[21] Cabañero-Martínez M.J., Cabrero-García J., Richart-Martínez M., Muñoz-Mendoza C.L. (2009). The Spanish Versions of the Barthel Index (BI) and the Katz Index (KI) of Activities of Daily Living (ADL): A Structured Review. Arch. Gerontol. Geriatr..

[22] Lawton M.P., Brody E.M. (1969). Assessment of Older People: Self-Maintaining and Instrumental Activities of Daily Living. Gerontologist.

[23] Gutiérrez-Valencia M., Leache L., Saiz L.C. (2022). Revisión de La Validez de Las Escalas de Valoración Del Riesgo de Caídas En Pacientes Hospitalizados. Rev. Esp. Geriatr. Gerontol..

[24] Aranda-Gallardo M., Morales-Asencio J.M., Canca-Sánchez J.C., Morales-Fernández Á., Moya-Suarez A.B., Mora-Banderas A.M., Pérez-Jiménez C., Barrero-Sojo S. (2015). Consequences of Errors in the Translation of Questionnaires: Spanish Version of Downton Index. Rev. Calid. Asist..

[25] Boletin Oficial del Esstado (2006). Ley 39/2006, de 14 de Diciembre, de Promoción de la Autonomía Personal y Atención a las Personas en Situación de Dependencia, Spain. https://www.boe.es/eli/es/l/2006/12/14/39.

[26] Glans M., Kragh Ekstam A., Jakobsson U., Bondesson Å., Midlöv P. (2020). Risk Factors for Hospital Readmission in Older Adults within 30 Days of Discharge—A Comparative Retrospective Study. BMC Geriatr..

[27] Martínez M.A.M., Alférez R.C., Mayor E.E., Blázquez M.R., Santamera A.S. (2011). Factores Asociados a Reingresos Hospitalarios En Pacientes de Edad Avanzada. Aten. Primaria.

[28] Contel J.C., Muntané B., Camp L. (2012). La Atención al Paciente Crónico en Situación de Complejidad: El Reto de Construir un Escenario de Atención Integrada. Aten. Primaria.

[29] Tavera-Vilchis M.A., Ruíz-Mauricio L.Y. (2023). Teorías de La Complejidad: Una Mirada Desde La Medicina Familiar. Arch. Med. Fam..

[30] Park M., Giap T.-T.-T., Lee M., Jeong H., Jeong M., Go Y. (2018). Patient- and Family-Centered Care Interventions for Improving the Quality of Health Care: A Review of Systematic Reviews. Int. J. Nurs. Stud..

[31] Salari N., Darvishi N., Ahmadipanah M., Shohaimi S., Mohammadi M. (2022). Global Prevalence of Falls in the Older Adults: A Comprehensive Systematic Review and Meta-Analysis. J. Orthop. Surg. Res..

[32] Zabalegui A., Juandó C., de Ormijana A.S., Ramírez A., Pulpón A., López L., Jones C., Izquierdo M.D., Gual P., González A. (2007). El Cuidador Informal de Personas Mayores de 65 Años en España. Rev. Rol. Enferm..

[33] Sánchez M., Rita M., Hernández García E.L., Medina Pérez M., Gómez Perera M., Estrada Suárez Pérez L., Navarro Vázquez F.J., Pérez F. (2014). Perfil del Cuidador Principal en el Área de Salud de Gran Canaria. Ene.

[34] Hanzeliková A., López-Muñoz F., Fusté-Moreno R. (2017). Perfil Socio-Demográfico de los Cuidadores de los Pacientes Geriátricos Hospitalizados Mayores de 75 Años y su Relación con la Satisfacción. Enferm. Glob..

[35] De la Fuente Robles Y., Morales E.S. (2015). The Spanish Long-Term Care System in the European Context. Arbor.

[36] Cantero-Garlito P.A., Flores-Martos J.A., Moruno-Miralles P. (2020). Dependency and Care: Perspectives from the Point of View of Professionals Assessing Situations of Dependency in Spain. Int. J. Environ. Res. Public Health.

[37] Barrio Cortes J., Suárez Fernández C., Bandeira de Oliveira M., Beca Martínez M.T., Lozano Hernández C., del Cura González I. (2020). Utilización de los Servicios de Salud de Atención Primaria en los Pacientes Crónicos según Nivel de Riesgo. Rev. Esp. Salud Publica.

[38] Glasinovic A., Canessa J., Sancy D., Sotomayor F. (2021). Buenas Prácticas en la Visita Domiciliaria Integral en Atención Primaria Chilena. Rev. Médica Clínica Las. Condes.

[39] Limón E., Riera N. (2023). Longitudinalidad y Continuidad En Atención Domiciliaria. Aten. Primaria.

[40] Lofti Fatemi N., Karimi Moonaghi H., Heydari A. (2019). Perceived Challenges Faced by Nurses in Home Health Care Setting: A Qualitative Study. Int. J. Community Based Nurs. Midwifery.

[41] Ocampo J.M., Reyes-Ortiz C.A. (2016). Revisión Sistemática de Literatura: Declinación Funcional en Ancianos Hospitalizados. Rev. Médica Risaralda.

[42] Fortinsky R.H., Covinsky K.E., Palmer R.M., Landefeld C.S. (1999). Effects of Functional Status Changes before and during Hospitalization on Nursing Home Admission of Older Adults. J. Gerontol. Ser. A Biomed. Sci. Med. Sci..

[43] Ocampo-Chaparro J.M., Reyes-Ortiz C.A. (2021). Efecto Conjunto de Deterioro Cognitivo y Condición Sociofamiliar sobre el Estado Funcional en Adultos Mayores Hospitalizados. Rev. Latinoam. Psicol..

[44] Jiménez Carrillo M., Martín Roncero U., Aldasoro Unamuno E., Morteruel Arizcuren M., Baza Bueno M. (2022). Percepciones y Experiencias de la Población ante la Transformación de la Modalidad de las Consultas en Atención Primaria durante la Pandemia. Aten. Primaria.

[45] Haines A., Pollock A., Victora C. (1971). 50 Years of the Inverse Care Law. Lancet.

